# Inhibiting FAK–Paxillin Interaction Reduces Migration and Invadopodia-Mediated Matrix Degradation in Metastatic Melanoma Cells

**DOI:** 10.3390/cancers13081871

**Published:** 2021-04-14

**Authors:** Antoine Mousson, Marlène Legrand, Tania Steffan, Romain Vauchelles, Philippe Carl, Jean-Pierre Gies, Maxime Lehmann, Guy Zuber, Jan De Mey, Denis Dujardin, Emilie Sick, Philippe Rondé

**Affiliations:** 1 Université de Strasbourg, CNRS UMR7021, Laboratoire de Bioimagerie et Pathologies, Migration, Invasion et Microenvironnement, Faculté de Pharmacie, 67401 Illkirch, France; antoine.mousson@unistra.fr (A.M.); marlene.legrand@unistra.fr (M.L.); tania.steffan@unistra.fr (T.S.); philippe.carl@unistra.fr (P.C.); jean-pierre.gies@unistra.fr (J.-P.G.); maxime.lehmann@unistra.fr (M.L.); jan.de-mey@unistra.fr (J.D.M.); denis.dujardin@unistra.fr (D.D.); emilie.sick@unistra.fr (E.S.); 2 Université de Strasbourg, CNRS UMR7021, Laboratoire de Bioimagerie et Pathologies, Plateforme PIQ, Faculté de Pharmacie, 67401 Illkirch, France; romain.vauchelles@unistra.fr; 3 Université de Strasbourg, CNRS UMR7242, Intervention Chémobiologique, ESBS, 67412 Illkirch, France; guy.zuber@unistra.fr

**Keywords:** FAK, tyrosine kinase inhibitor, paxillin, invadopodia, migration, melanoma

## Abstract

**Simple Summary:**

The focal adhesion kinase (FAK) is over-expressed in a variety of human tumors and is involved in many aspects of the metastatic process. This has led to the development of small inhibitors of FAK kinase function which are currently evaluated in clinical trials. We demonstrate here that this class of inhibitors, while decreasing melanoma cell migration, increases invadopodia activity in metastatic melanoma cells. Searching for an alternative strategy to inhibit the oncogenic activity of FAK, we show that inhibiting FAK scaffolding function using a small peptide altering FAK–paxillin interactions reduces both migration and invadopodia-mediated matrix degradation in metastatic melanoma cells.

**Abstract:**

The nonreceptor tyrosine kinase FAK is a promising target for solid tumor treatment because it promotes invasion, tumor progression, and drug resistance when overexpressed. Investigating the role of FAK in human melanoma cells, we found that both in situ and metastatic melanoma cells strongly express FAK, where it controls tumor cells’ invasiveness by regulating focal adhesion-mediated cell motility. Inhibiting FAK in human metastatic melanoma cells with either siRNA or a small inhibitor targeting the kinase domain impaired migration but led to increased invadopodia formation and extracellular matrix degradation. Using FAK mutated at Y397, we found that this unexpected increase in invadopodia activity is due to the lack of phosphorylation at this residue. To preserve FAK–Src interaction while inhibiting pro-migratory functions of FAK, we found that altering FAK–paxillin interaction, with either FAK mutation in the focal adhesion targeting (FAT) domain or a competitive inhibitor peptide mimicking paxillin LD domains drastically reduces cell migration and matrix degradation by preserving FAK activity in the cytoplasm. In conclusion, our data show that targeting FAK–paxillin interactions could be a potential therapeutic strategy to prevent metastasis formation, and molecules targeting this interface could be alternative to inhibitors of FAK kinase activity which display unexpected effects.

## 1. Introduction

Melanoma is a very aggressive form of skin cancer which accounts for more than 80% of skin cancer-related deaths. The prognosis in melanoma is directly proportionate to the depth of the neoplasm, and patients with spreading melanoma have a very poor prognosis with a five-year survival rate of less than 5%. The spreading of melanoma is a multistep process which involves several genetic alterations [1]. In melanoma, as in many invasive epithelial cancers, such as head, neck, breast, or prostate, cancer cells acquire the ability to breach the basement membrane using supramolecular complexes called invadopodia. Invadopodia are sub-cortical protrusions that localize matrix-degrading activity to cell-substrate contact points and represent major hubs where many signaling pathways converge [2]. Invadopodia are composed of an actin-rich core that includes actin activators, nucleators, and regulators surrounded by adhesion, scaffolding, and signaling proteins [3]. These structures concentrate proteolytic enzymes such as matrix metalloproteinases (MMPs), thus mediating extracellular matrix (ECM) degradation. Indeed, MMP are upregulated in invasive melanoma and there is extensive evidence that they have a role in promoting the dissemination of melanoma [4].

Tumoral cell invasion is a highly complex process that requires both cell-degrading capabilities to breach the basement membrane and enter the circulation, and cell adhesion and migration to the environing substratum. In mesenchymal-like cells, cells formed focal adhesion (FA) by clustering at sites of integrin engagement to the ECM, structural, scaffolding and signaling proteins. Among them, the focal adhesion kinase (FAK) is a crucial signaling protein that integrates signals from integrins to the actin filaments, thereby controlling FA turn-over which tunes the migration speed [5,6]. This control is made in part by phosphorylation and dephosphorylation processes [7,8]. Structurally, FAK is a 125 kDa protein that contains an N-terminal FERM domain, a central kinase domain, and a C-terminal domain which contains the focal adhesion targeting (FAT) site [9]. Upon different stimuli including integrin clustering, the autophosphorylation of FAK at Y397 creates a binding site for Src, which can phosphorylate other tyrosines on the FAK sequence, thus creating new binding sites for SH2 domain-containing proteins. The activated FAK/Src complex also phosphorylates numerous downstream targets with roles in cell survival, proliferation, migration, and invasion. The precise role of FAK in invasion has been subject to controversy. The current consensus is that FAK does not localize to invadopodia but seems to regulate their formation and activity from a distance by controlling the localization of the Src kinase. Indeed, the depletion of FAK in melanoma and breast cancer cells releases Src from FA, thereby activating invadopodia targets and promoting invadopodia activity [10,11,12]. Whether FAK inhibitors currently under development display similar unexpected effects remains to be determined. Nevertheless, in melanoma, it was reported that FAK promotes an aggressive phenotype which may correlate with its role in promoting cell migration [13].

Given the panel of FAK downstream signaling effectors, it is not surprising that this protein is overexpressed in many tumors and is associated with tumor growth, invasion, and resistance to treatment. Thus, the development of FAK antagonists, as anticancer therapy, led to several small inhibitors of FAK kinase function. For example, PF-573,228 inhibits FAK phosphorylation on Tyr397 and reduces both chemotactic and haptotactic migration, concomitant with FA turnover inhibition [14]. FAK kinase inhibitors currently undergoing clinical trials are almost all small ATP-competitive molecules which inhibit the *trans*-autophosphorylation of FAK at tyr397, leading to altered Src recruitment. Nonetheless, besides its kinase function, FAK also possesses scaffolding functions highly relevant in cancer signaling. Indeed, as a multi-domain protein, FAK can act as an assembly platform for protein complexes to bridge proteins. These complexes and bridges may serve as targets for the development of new FAK inhibitors based on protein–protein interactions. Protein–protein interaction inhibitors (PPIIs) are today an attractive novel tool for cancer therapy, and several reports have described PPIIs for FAK, targeting either the p53 binding site on the FERM domain [15] or the VEGFR-3 binding site on the FAT domain [16]. Moreover, some of the compounds developed for these targets showed interesting anti-tumor activity in xenograft mouse models.

In this study, we demonstrate that inhibiting FAK with either siRNA or a small inhibitor targeting the kinase domain while decreasing melanoma cell migration led to increased invadopodia activity in metastatic melanoma, but not in in situ melanoma. Using mutation of FAK at Tyr397, a binding site for Src, we confirmed that this mutation while reducing cell migration, releases a pool of active Src that mediates enhanced invadopodia activity. Searching for alternative FAK inhibition strategies that preserve FAK/Src binding, we found that altering FAK–paxillin interaction, with either a FAK mutation at its paxillin binding site or a peptide that disturbs FAK–paxillin interaction, drastically reduces cell migration and matrix degradation. Taken together, our data provide a new horizon for the development and use of FAK PPIIs.

## 2. Materials and Methods

### 2.1. Antibodies

For immunofluorescence, anti-FAK (610088) was obtained from BD; anti-cortactin (05-180) from Millipore; anti-phospho-Y118 paxillin (44-722G), anti-paxillin (AHO0492) and anti-phospho-Y397 FAK (44-625G) from Invitrogen; phalloidin 568 (A12380), phalloidin 647 (A22287) and Alexa Fluor-conjugated secondary antibodies from Molecular Probes. For Western blotting and co-immunoprecipitation, anti-phospho-Y397 FAK (44-625G), anti-paxillin (AHO0492), was obtained from Invitrogen; eGFP (66002-1-IG) from Proteintech anti-FAK (610088) from BD; anti-b-actin (MA1115) from Boster; and HRP conjugated secondary antibodies from Biorad.

### 2.2. Cells Culture

Normal human primary epidermal melanocytes, from Lonza, were cultivated in MGM-4 medium (PS-200-041, ATCC). Primary human melanoma cell lines were purchased from Rockland and cultured in MCDB153/L-15 medium (M7403, Sigma, St. Louis, MO, USA; 11415064, Invitrogen) supplemented with 2% fetal bovine serum, 1.68 mM CaCl_2_, and 1% penicillin/streptomycin. The A375 melanoma cell line from ATCC was maintained in DMEM (11960044, Lonza, Basle, Switzerland) supplemented with 10% fetal bovine serum, 1 mM L-glutamine, and 1% penicillin/streptomycin antibiotics.

### 2.3. siRNA Transfections and Cells Treatments

Melanoma cells were plated at 2.5 × 10^5^ cells in six-well plates, transfected with 50 pmol of siRNA targeting the 5′-UTR regions of FAK as previously described [12], and incubated for 24 h at 37 °C before use. For 3D collagen matrix assay, cells were treated with 10 µM of the metalloprotease inhibitor GM6001 (S7157, Selleckchem). For Western blot and matrix degradation assays, cells were treated with 1 µM of the FAK inhibitor PF-573228 (S2013, Selleckchem, Houston, TX, USA) for 12 h. For the wound healing assay, melanoma cells were treated with mitomycin C (S8146, Selleckchem) for 2 h at 10 µg/mL before the scratch to inhibit cell replication and with PF-573228 at 1 µM.

### 2.4. Cells Transfections

FAK-WT-GFP, FAK-Y397F-GFP and FAK-I936E I998E-GFP were obtained as previously described [17]. The LD2-LD4-GFP sequence of paxillin (aa 136–296) was inserted into the pEGFP–C1 plasmid (Clontech, Kusatsu City, Japan), creating a fusion of enhanced GFP (EGFP) to the N terminus of paxillin. Melanoma cells were co-transfected with siRNA and 4 µg FAK WT or FAK mutant DNA or with LD2-LD4-GFP DNA 4 µg using lipofectamine 2000 (Invitrogen, Carlsbad, CA, USA), according to the manufacturer’s instructions. Transfected cells were incubated for 24 h at 37 °C before use.

### 2.5. 3D Collagen Matrix Assay

The invasion assay was adapted from Sadok et al. [18]. Cells lines labelled with Hoechst 33,342 were suspended in 3 mg/mL of serum-free solution of collagen Bovine Type I neutralized (5005-B, Advanced biomatrix) at 3 × 10^5^ cells/mL. Then, 600 µL were dispensed into black 24-well plates (058062, Dutscher) coated with bovine serum albumin. The plates were centrifuged at 1500 rpm at 4 °C for 5 min and incubated at 37 °C for 3 h. Once collagen had polymerized and the medium was supplemented with 10% fetal bovine serum, 100 ng/mL EGF with either drug was added on top of the collagen. After 24 h, cells were observed using a Leica TSC SPE confocal microscope (×20 HCX Pl Apo 0.7 NA objective, Wetzlar, Germany) and z-stacks were acquired with a step of 1 µm in four fields per sample. Nuclear localization was quantified by Imaris at each plane. The invasion index was calculated by reporting the number of cells above 10 µM on the total number of cells by field.

### 2.6. Matrix Degradation Assay and Immunofluorescence

Glass-bottom Ibidi dishes (81158, Ibidi) were coated with fluorescent-labelled-FITC gelatin or Cy3 gelatin as described previously [12]. Then, 3.5 × 10^4^ previously starved melanoma cells were plated on fluorescent gelatin and incubated at 37 °C for 24 h in medium supplemented with 10% fetal bovine serum. Cells were fixed using 4% paraformaldehyde permeabilized using triton-X-100 at 0.1% and incubated in 2% of bovine serum albumin at room temperature. Cells were then labelled for 1 h with anti-cortactin (1/250), anti-phospho-Y397 FAK (1/100), anti-FAK (1/250), anti-phospho-Y118 paxillin (1/250) or anti-paxillin (1/250). After three washes with PBS, cells were incubated with Alexa-488-conjugated goat anti-rabbit (1/1000), Alexa-647-conjugated goat anti-rabbit (1/1000), Alexa-405-conjugated goat anti-mouse (1/1000), Alexa-647-conjugated goat anti-mouse (1/1000), Alexa-568-conjugated phalloidin (1/250) or Alexa-647-conjugated phalloidin (1/250) for 1 h, washed, and mounted in Prolong Gold mounting media (Invitrogen). Cells were imaged using a Leica TSC SPE confocal microscope (×63 HCX Pl Apo 1.40 NA or ×20 HCX Pl Apo 0.7 NA objective, Wetzlar, Germany). Invadopodia were identified as actin- and cortactin-rich punctate structures. Areas of degradation were identified as “black holes” within the fluorescent gelatin. Invadopodia and areas of degradations were quantified using ImageJ software. Degradation areas measurements were based on cells displaying degradation activity, and the frequency of degradation was based on randomly selected cells.

### 2.7. Wound Healing

Melanoma cells were seeded in 12-well plates containing medium supplemented with 10% fetal bovine serum and transfected with siCtrl or siFAK as described before. After 24 h, confluent cell layers were treated with mitomycin C and with PF-573228 as described before and then wounded with a pipette tip. Images of cell migration were acquired using a Leica DMIRE2 microscope (×10 N PLAN 0.25 NA objective) equipped with a 37 °C 5% CO_2_ control system (Life Imaging Services) with a Leica DC350FX CCD camera piloted by FW4000 software (1 image every 30 min for 24 h).

### 2.8. Co-Immunoprecipitation and Western Blot

For co-immunoprecipitation, A375 melanoma cells were seeded in 90 mm Petri dishes and co-transfected as previously described with siFAK, siFAK and FAK-GFP, or siFAK and FAKI936E/I998E-GFP. After 48 h, cells were washed twice with ice-cold PBS and lysed with modified RIPA buffer (137 mM NaCl, 20 mM Tris, pH 8.0, 10% glycerol, 1% IGEPAL CA-630, 3 mM Na_3_VO_4_) complemented with a protease and phosphatase inhibitor cocktail (78441, Invitrogen). Lysates were centrifuged at 15,000× *g* for 15 min at 4 °C. Then, 500 µg of cell lysates were incubated with specific anti-FAK antibody (1/100) for 3 h at 4 °C with continuous shaking. Protein G sepharose beads (GE Healthcare) were then added for overnight incubation. Beads were then washed 3 times with ice-cold RIPA buffer and resuspended in Laemmli buffer.

For Western blots, total cell proteins were obtained by lysing cells for 15 min at 4 °C in RIPA buffer (8990, Invitrogen) supplemented with a protease and phosphatase inhibitor cocktail (78441, Invitrogen). Lysates were centrifuged at 15,000× *g* for 15 min at 4 °C. Protein concentration was determined using a DC Protein Assay Kit (BioRad, Hercules, CA, USA). Then, 20 mg of proteins were suspended in Leammli buffer and loaded on 8% polyacrylamide gel, and transferred to a PVDF membrane. Non-specific sites were blocked for 1 h at room temperature using 5% BSA in TBS-T (pH 7.4, 10 mM Tris, 150 mM NaCl and 0.1% Tween 20).

After that, membranes were incubated with primary antibody diluted in TBS-T containing 5% BSA or 5% non-fat milk overnight at 4 °C. After washing membranes three times in TBS-T for 5 min, specific horseradish peroxidase-labeled secondary antibodies were incubated with membranes for 1 h at room temperature. Signals were detected using an ECLplus kit (170-5061, Biorad). Bands were quantified using ImageQuantTL (GE Healthcare).

### 2.9. Statistical Analysis

Statistical significance was calculated using an unpaired, two-tailed Student’s *t*-test. Values were considered statistically significant when the *p*-value was <0.05. For all figures: *, *p* < 0.05; **, *p* < 0.01; ***, *p* < 0.001. Error bars represent SD.

## 3. Results

### 3.1. Aggressivity of Melanoma Cells is Associated with Invadopodia-Mediated Proteolytic Potential

**Figure 1:**
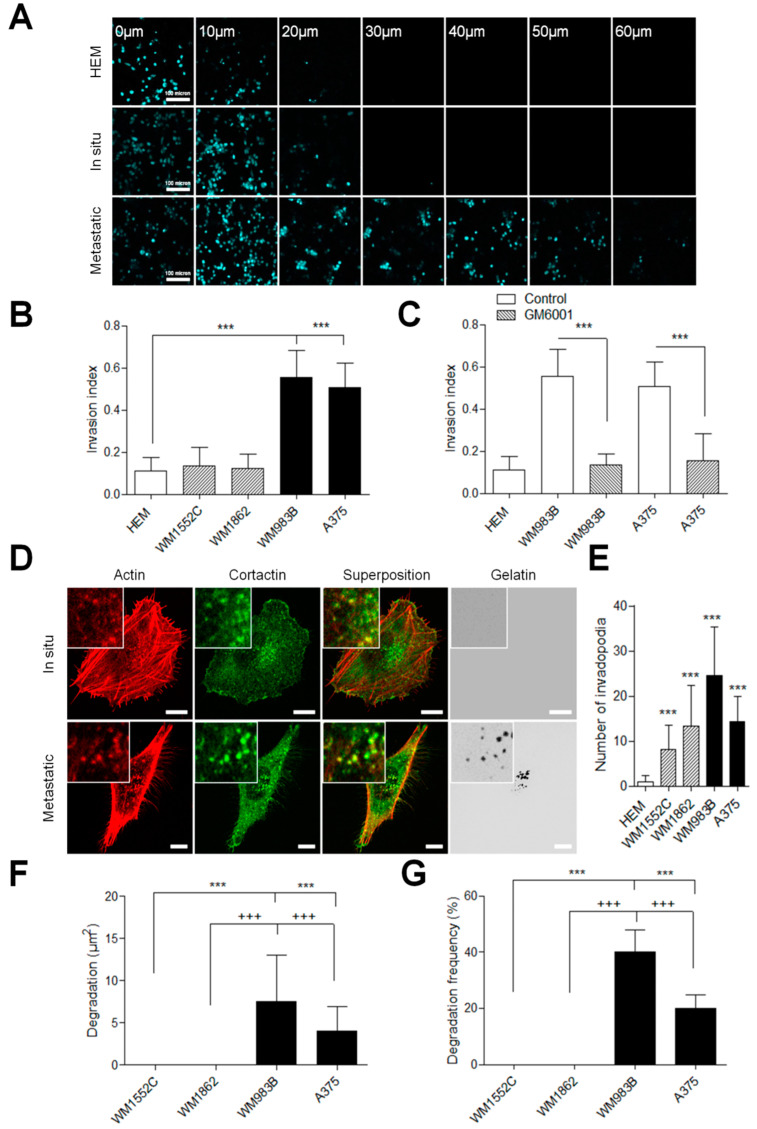
Invasive potential of melanoma cells is matrix metalloproteinase (MMP)-dependent and regulated by invadopodia activity. (**A**) Melanoma cell lines labelled with Hoechst 33,342 were assessed for invasion into collagen-I over 24 h. Representative images of human epidermal melanocytes (HEM), WM1862 and WM983B melanoma cells in control condition between 0 and 60 µm. Scale bar: 100 µm. The invasion index was calculated by reporting the number of cells above 10 µm on the total number of cells by the field (**B**) in control conditions, or (**C**) in the presence of the non-selective MMP inhibitor, GM6001. Histograms represent the mean ± SD from 3 independent experiments. ***, *p* < 0.001; unpaired *t*-test compared to HEM or non-treated cells. (**D**) Melanoma cell lines were plated on FITC–gelatin (gray), fixed, and labelled for actin (red) and cortactin (green). Boxed regions and insets depict the colocalization of actin and cortactin (yellow) as markers for invadopodia. Degradation was identified as black holes on fluorescent gelatin. Scale Bar: 10 µm. (**E**) Histograms represent the mean ± SD of invadopodia numbers per cell (*n* = 56–109). (**F**) Histograms represent the mean ± SD of the area of degradation (*n* = 40−142), and of the degradation frequency (**G**) from at least 3 independent experiments. ***, *p* < 0.001; unpaired *t*-test compared to * WM1552C or †WM862.

Gaining invasiveness is one of the first and most critical steps of metastatic dissemination. The prognosis for patients with melanoma significantly worsens with more profound invasion levels [19]. To get insight into the mechanism driving invasion, we tested a panel of melanoma cell lines for their ability to invade a 3D collagen matrix. In situ melanoma cell lines showed a progression similar to normal human melanocyte (HEM), whereas malignant melanoma cell lines progressed more efficiently into the collagen matrix (Figure 1A). Indeed, the index of invasion of metastatic melanoma cells was increased compared to the primary melanoma, suggesting improved invasive properties during the melanoma progression (Figure 1B). Efficient invasion potential was due to an increase in metalloprotease activity, as demonstrated upon treatment of melanoma cells with the metalloprotease inhibitor GM6001 that completely abolished the ability of metastatic melanoma cells to migrate into the 3D collagen matrix (Figure 1C). Altogether, these data suggest that proteolytic activity regulates the aggressivity potential of melanoma cell lines.

Invadopodia are specialized cellular structures present in cells with pathological invasive behaviors [20]. To elucidate the mechanism implicated in the melanoma cell’s invasive potential, we plated cells on fluorescent gelatin and quantified the invadopodia number and activity on cell lines. Surprising, invadopodia, identified as actin- and cortactin-rich punctate structures, were present in all melanoma cell lines, including in situ melanoma with no apparent link between the number of invadopodia per cell and their respective invasive potential in the collagen matrix (Figure 1D,E). Next, we analyzed invadopodia activity by measuring their matrix-degrading capabilities. Our results showed that all metastasis cell lines could degrade the fluorescent gelatin in opposition to the in situ melanoma cells studied (Figure 1F). This difference was, however, not due to a change in MT1-MMP expression (Figure S1) Furthermore, degradation frequency based on random fields’ observation showed that degradation is absent in in situ cell lines, in clear opposition to the metastatic cells which displayed a degradation frequency from 20% to 40.1% (Figure 1G). Altogether, these results support the idea that invadopodia activities regulate the aggressiveness of melanoma cell lines.

### 3.2. FAK Expression is Increased But Not Correlated with Melanoma State

**Figure 2:**
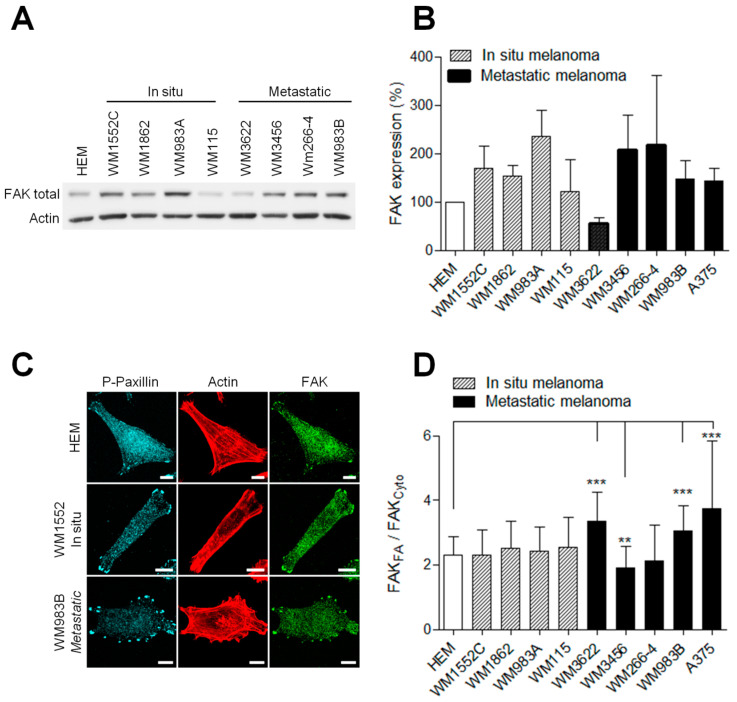
FAK expression is increased in melanoma cells. (**A**) Representative Western blot of HEM and melanoma cells. Cellular extracts were analyzed by Western blotting and probed for total FAK and actin as a loading control. (**B**) Histograms represent the mean ± SD from 3 independent experiments (**C**) Melanoma cell lines were immunostained for P-Y118-paxillin (cyan), actin (red) and FAK total (green). Scale bar: 10 µm. FAK localizations in melanoma cells (*n* = 46–70) were measured by reporting the FAK signal in FA over FAK signals in the cytoplasm. (**D**) Histograms represent the mean ± SD from 3 independent experiments. **, *p* < 0.01; ***, *p* < 0.001; unpaired *t*-test compared to HEM.

FAK has been described to control many aspects of the metastatic process, including cell migration and invasion [21]. Moreover, FAK overexpression has been reported in various human cancers [22,23]. Therefore, to investigate the role of FAK in melanoma progression, we first compared FAK expression level in normal human epidermal melanocytes (HEM) and a panel of in situ and malignant human melanoma cell lines (Figure 2A and Figure S2). We observed that FAK was overexpressed in the majority of melanoma cells compared to the normal human epidermal melanocyte. However, no apparent link between the FAK level and the tumor grade was observed (Figure 2B), corroborating clinical studies from Ricker et al. from the Oncomine cancer dataset. As FAK localization greatly affects its functions [17,24], we further compared its subcellular localization by immunofluorescence in our different cell models (Figure 2C). We noticed a striking accumulation of FAK at focal adhesions identified by phospo-Y118-paxillin and weaker FAK staining in the cytoplasm. Quantification revealed that FAK enrichment at FAs, identified by P-paxillin staining, increased by 66% in metastatic melanoma cells compared to HEMs (Figure 2D). However, there was no correlation between total FAK expression level or FAK accumulation at FAs and the migration or the degradation properties of melanoma cells as previously described.

### 3.3. FAK Inhibition Reduces Migration but Increases Proteolytic Activity of Invasive Melanoma Cells

**Figure 3:**
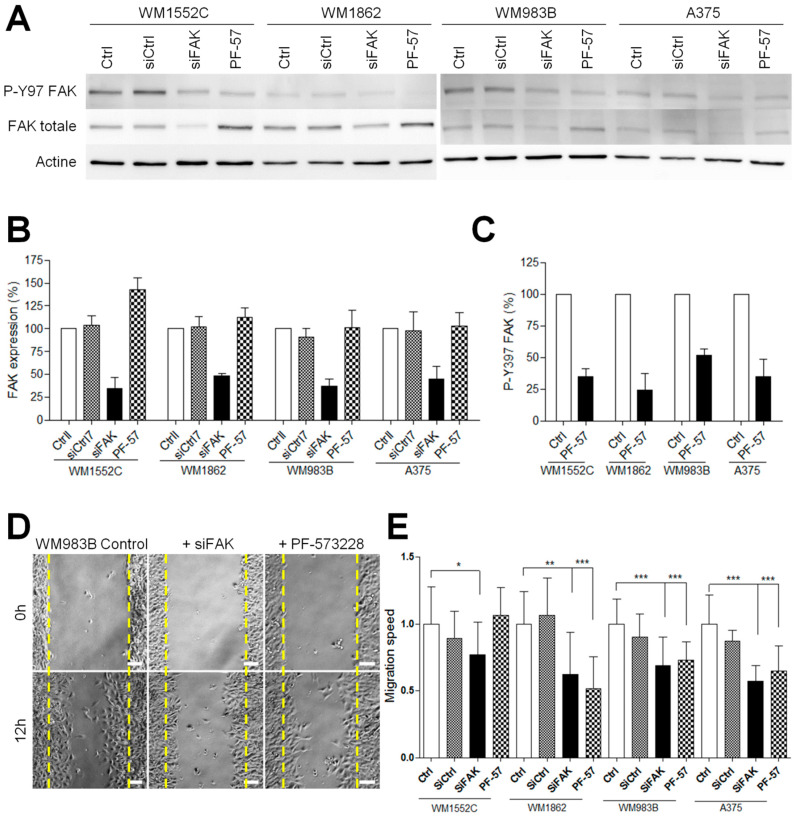
FAK inhibition reduces the migration of invasive melanoma cells. (**A**) Melanoma cells were transfected for 48 h with control siRNA or FAK siRNA or were treated by PF-573228 at 1 µM for 12 h. Cellular extracts were analyzed by Western blotting and probed for P-Y397 FAK, FAK total, and actin as a loading control. (**B**) Quantification of FAK expression and (**C**) FAK activity after siRNA transfection or PF-573328 from 3 independent experiments. (**D**) Confluent cell layers of melanoma cell lines transiently transfected with control siRNA or FAK siRNA or treated by PF-573228 were wounded and cells were allowed to migrate for 12 h. Images represent WM983B melanoma cells at 0 h and 12 h. (**E**) Histogram represent the mean speed migration normalized to control ± SD from at least 3 independent experiments *, *p* < 0.05; **, *p* < 0.01; ***, *p* < 0.001; unpaired *t*-test compared to control condition.

**Figure 4:**
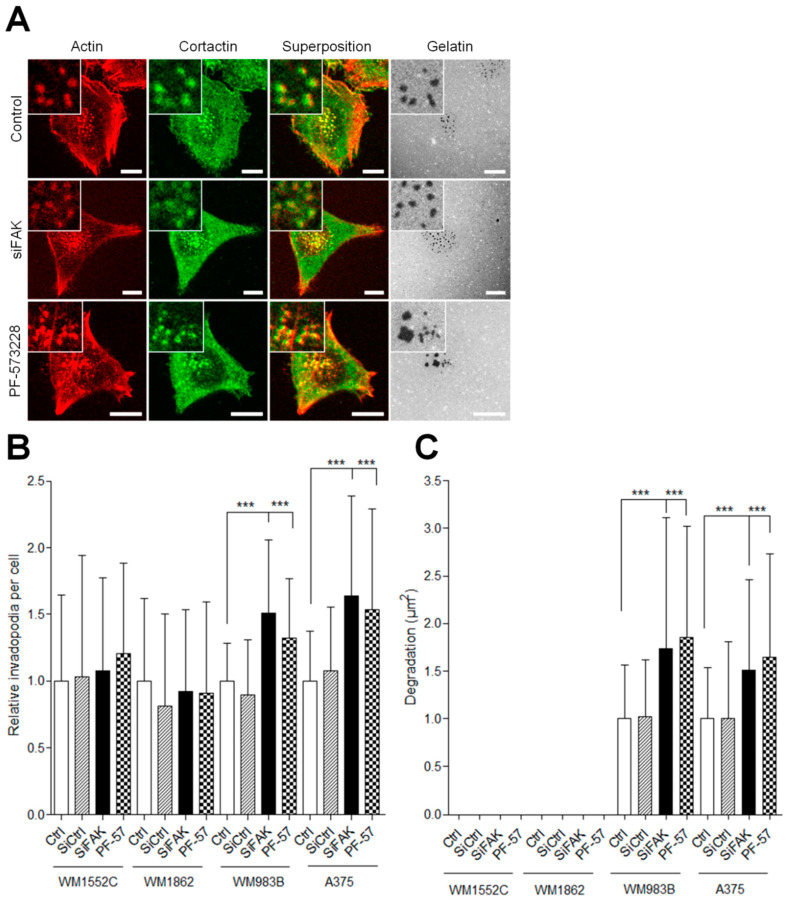
FAK inhibition increases the proteolytic activity of invasive melanoma cells. (**A**) Melanoma transiently transfected or treated by PF-573228 were plated on FITC–gelatin (gray), fixed, and labelled for actin (red) and cortactin (green) Scale bar: 10 µm. Histograms represent the mean normalized to control ± SD of (**B**) invadopodia per cell (*n* = 33–59) and (**C**) area of degradation (*n* = 48–60) from 3 independent experiments ***, *p* < 0.001; unpaired *t*-test compared to control condition.

To investigate the role of FAK during invasion, we compared the ability of metastatic and in situ melanoma cell lines to degrade the extracellular matrix after siRNA-mediated FAK silencing [12] or after treatment with the FAK tyrosine kinase inhibitor PF-573228 [14]. Western blot analysis (Figure 3A) revealed that FAK knockdown efficiency reached 52 to 66% (Figure 3B and Figure S3). On the other hand, PF-573228, inhibited between 50 and 75% of FAK activity after 12 h of treatment at 1 µM (Figure 3C and Figure S3). Surprisingly, FAK expression level was increased in WM1552C melanoma cell lines after treatment with PF-573228 (Figure 3B). We next investigated whether FAK depletion or inhibition affects melanoma cell migration using the wound healing assay. We observed that FAK depletion or inhibition reduced the migration speed by 30–50% compared to control conditions, as already demonstrated in various cancers cell lines [25,26] (Figure 3D,E). These results confirmed that FAK expression is necessary for optimal melanoma cell migration independently of melanoma aggressivity. Interestingly, treatment with PF-573228 inhibited migration speed in all cell lines studied except WM1552C cells, which is probably related to the upregulation of FAK in this cell line. As melanoma progression in a 3D matrix requires invadopodia activity, we further analyzed the effect of FAK depletion or inhibition on invadopodia properties (Figure 4A). Surprisingly, we show that knockdown of FAK in metastatic melanoma cells significantly increased the number of invadopodia per cells and the occurrence of matrix degradation compared to control condition (Figure 4B,C), confirming previous studies in breast cancer cells [10,11]. Similar results were obtained in metastatic melanoma cells treated with the FAK kinase inhibitor PF-573228. However, FAK depletion or FAK inhibition with PF-573228 did not affect the number of invadopodia or their activity in in situ melanoma cells (Figure 4B,C). Altogether, these data suggest that the kinase activity of FAK inhibits invadopodia formation and activity in the more aggressive stages of melanoma development.

### 3.4. Inhibiting FAK/Src Interaction Increases Invadopodia Activity

**Figure 5:**
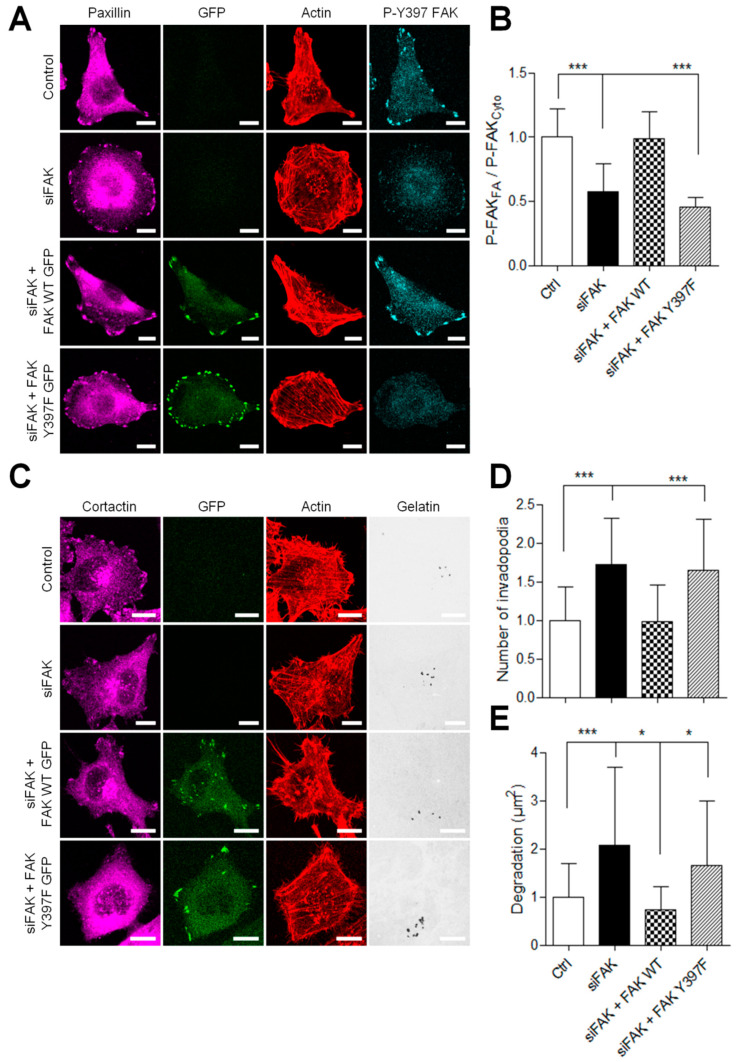
Inhibiting the FAK–Src interaction increases the proteolytic activity of invasive melanoma cells. (**A**) A375 melanoma cells transiently co-transfected with siFAK and wild-type FAK-GFP or FAK-Y397F-GFP were fixed and labelled for paxillin (purple), actin (red) and P-Y397 FAK (cyan). (**B**) Histogram represents the mean normalized to control ± SD of P-FAK localization in melanoma cells. Signals were measured by reporting the P-FAK signal in FA over that in the cytoplasm from 3 independent experiments (**C**) A375 melanoma cells cotransfected as described above were plated on Cy3-gelatin (gray), fixed, and labelled for actin (red) and cortactin (purple). Histograms represent the mean normalized to control ± SD of (**D**) invadopodia by cell (*n* = 40–62) and (**E**) area of degradation (*n* = 51–118) from 3 independent experiments. *, *p* < 0.05; ***, *p* < 0.001; unpaired *t*-test compared to the control condition. Scale bar: 10 µm

We and others have previously reported that FAK controls the tyrosine phosphorylation balance between focal adhesions and invadopodia by regulating Src localization [10,11,12]. Thus, inhibiting FAK–Src interactions would tip the balance toward invadopodia and therefore have similar effects as FAK inhibition. FAK phosphorylation at Tyr397 creates a binding site for Src via its SH2 domain. Therefore, to test this hypothesis, we transfected A375 melanoma cells with FAK-Y397F-GFP, a non phosphorylable form of FAK in cells depleted for FAK. As expected, in siFAK-treated cells, FAK phosphorylation at Tyr397 is strongly reduced (Figure 5A,B), whereas re-expression of FAK-WT-GFP but not FAK-Y397F-GFP rescued this effect. We next tested the functional impact of FAK-Y397F expression on invadopodia activity. As shown in Figure 5C–E, FAK depletion increases invadopodia formation and activity, whereas rescue with FAK-WT but not FAK-Y397F abolished this effect. The latter suggests that preserving the FAK–Src interaction via sustained FAK phosphorylation at Tyr397 would keep Src from activating downstream substrates at invadopodia.

### 3.5. Targeting FAK–Paxillin Interaction, an Alternative Strategy to Kinase Inhibitors

We next reasoned that to reduce melanoma aggressiveness, a molecule targeting FAK should inhibit FAK functioning at FAs, diminishing cell migration while at the same time preserving sufficient phosphorylation at Tyr397. Indeed, this would prevent the release of active Src from FAs, and thus FAK inhibition-mediated increased proteolytic activity. We have previously described that inhibiting FAK–paxillin interaction using the FAK mutation FAK-I936E/I998E reduces cell migration in vitro by a mechanism involving altered FAK localization at FAs [17]. Of importance, expression of this mutant in FAK null cells, while leading to reduced phosphorylation of FAK targets, retained substantial phosphorylation at its Tyr397 residue. Therefore, we examined the effect of this mutant on invasion by cotransfecting A375 melanoma cells with FAK siRNA and either wild-type FAK or FAK-I936E/I998E tagged with GFP.

**Figure 6:**
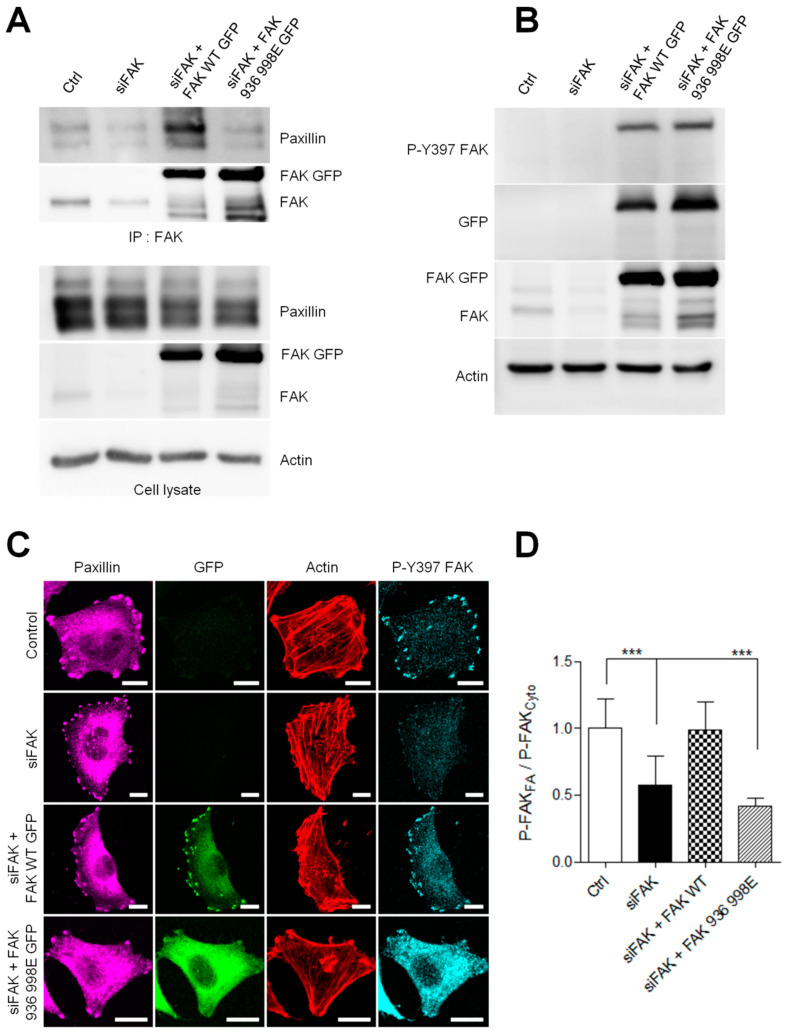
FAK–paxillin interaction conditions FAK localization to FA. (**A**) Cell extracts from A375 melanoma cells transiently co-transfected with siFAK and wild-type FAK-GFP or FAK-I936/I998-GFP were analyzed by immunoprecipitation (IP) using anti-FAK Ab and blotted for paxillin and FAK. The expression level of proteins in the corresponding cell lysate is shown. (**B**) Representative Western blot showing A375 melanoma cells transfected as described above and blotted for P-Y397 FAK, FAK total, eGFP and actin. (**C**) Cells transiently co-transfected with siFAK and wild-type FAK-GFP or FAK-I936/I998-GFP were fixed and labelled for paxillin (purple), actin (red) and P-Y397 FAK (cyan). Scale bar: 10 µm. (**D**) Histograms represent the mean normalized to control ± SD of FAK localization in melanoma cells. Signals were measured by reporting the P-FAK signal in FA over that in the cytoplasm from 3 independent experiments ***, *p* < 0.001; unpaired *t*-test compared to control condition.

First, we performed co-immunoprecipitation experiments to verify whether the FAK mutation at I936/I998 disrupts FAK–paxillin interaction. As shown in Figure 6A, in FAK-depleted cells, mutant FAK-I936/I998 was almost entirely unable to precipitate paxillin compared to wild-type FAK. To investigate the effects of I936/I998 mutations on FAK activation, we performed Western blotting to measure the state of FAK phosphorylation at its Y397 residue. No significant difference in FAK phosphorylation on this site was observed between FAK-I936/I998-GFP and FAK-WT-GFP transfected cells (Figure 6B). To visualize if these mutations altered FAK localization at FAs, we analyzed paxillin localization by immunofluorescence, a marker of FAs, in FAK-depleted A375 melanoma cells expressing FAK-WT-GFP or FAK-I936/I998-GFP. While FAs were clearly positive for paxillin staining, the FAK mutant, contrary to FAK-WT, was mainly localized into the cytoplasm and not at FAs, as shown by the distribution of GFP signal (Figure 6C).

Moreover, analyzing P-Y397 FAK staining, we observed a substantial accumulation of active FAK at FAs and a weak one in the cytoplasm in control cells (Figure 6C). As expected, in siFAK-treated cells, the quantification of active FAK revealed a 46% reduction in P-Y397 staining at FA compared to control cells. (Figure 6D). Re-expression of FAK-I936/I998 in FAK-depleted cells failed to rescue this phenotype and displayed an accumulation of active FAK in the cytoplasm and a weak signal at FA, thus decreasing the FA/cytoplasm ratio in close opposition to the high FA/cytoplasm ratio observed in the FAK WT rescue experiment. Altogether, these results confirm that inhibiting FAK–paxillin interaction delocalizes FAK from FAs. However, despite its cytoplasmic localization, FAK-I936/I998 preserves part of its activity as observed by the increased accumulation of cytoplasmic P-Y397 FAK, which may thus interact with partners such as Src displaying SH2 binding sites.

**Figure 7:**
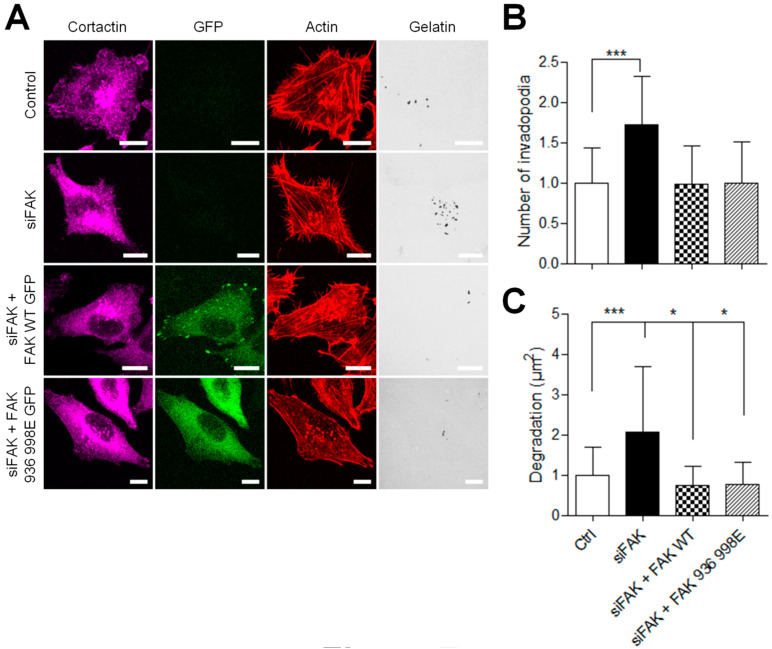
Inhibiting FAK–paxillin interactions reduces proteolytic activity. (**A**) A375 melanoma cells transiently co-transfected with siFAK and wild-type FAK-GFP or FAK-I936/I998-GFP were plated on Cy3-gelatin (gray), fixed, and labelled for actin (red) and cortactin (purple) Scale bar: 10 µm. Histograms represent the mean normalized to control ± SD of (**B**) invadopodia by cell (*n* = 50–62) and (**C**) area of degradation (*n* = 66–118) from 3 independent experiments. *, *p* < 0.05; ***, *p* < 0.001; unpaired *t*-test compared to control condition.

Next, to investigate whether the inhibition of FAK–paxillin interactions affects melanoma invasion, siFAK-treated A375 melanoma cells expressing wild-type FAK-GFP or FAK-I936/I998-GFP were plated on Cy3-gelatin and labeled for actin and cortactin (Figure 7A). As reported above, FAK depletion increases invadopodia formation and activity, whereas rescue with FAK-WT abolished this effect. More importantly, rescue with FAK-I936/I998 also abolished this effect; melanoma cells expressing FAK-I936/I998 displayed both reduced invadopodia number and area of degradation as compared to FAK siRNA-treated cells (Figure 7B,C). These results indicate that inhibiting FAK–paxillin interaction reduces the proteolytic activity of melanoma cells.

### 3.6. Disturbing FAK/Paxillin Interaction Reduces Invasion and Migration

**Figure 8:**
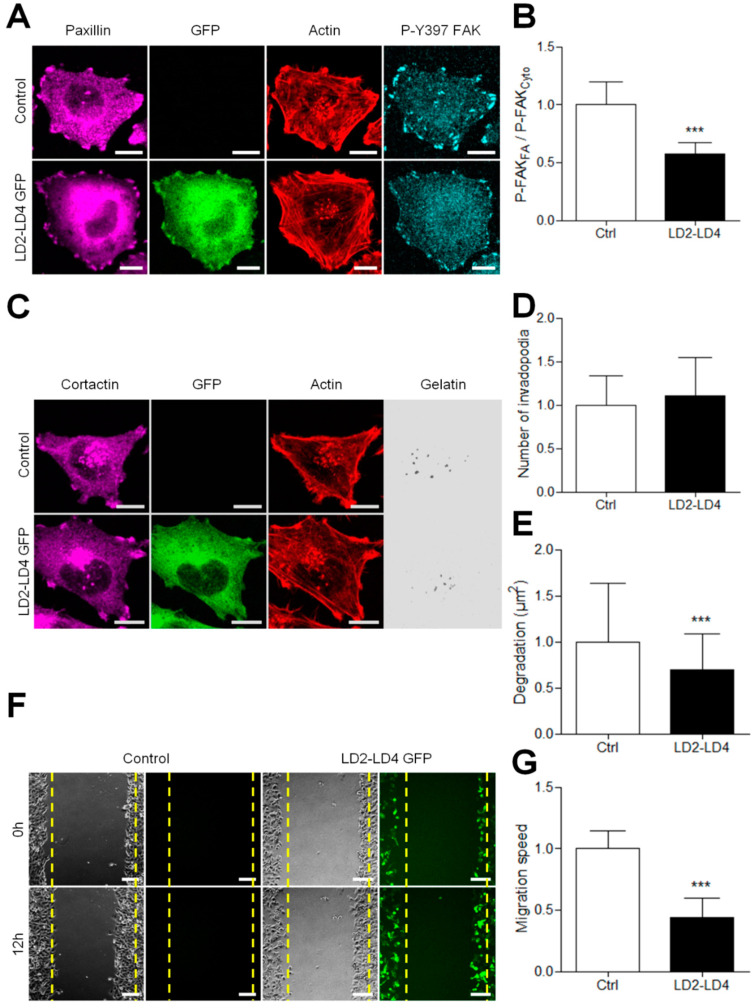
Competitively inhibiting FAK–paxillin interactions reduces both migration and degradation. (**A**) A375 melanoma cells transiently transfected with LD2–LD4 tagged with GFP were fixed and labelled for paxillin (purple), actin (red) and P-Y397 FAK (cyan) Scale bar: 10 µm. (**B**) Histogram represents the mean normalized to control ± SD of FAK localization in melanoma cells. Signals were obtained by reporting the FAK signal in FA on that in the cytoplasm from 3 independent experiments. (**C**) A375 melanoma cells transiently transfected with LD2–LD4 tagged with GFP were plated on Cy3-gelatin (gray), fixed, and labelled for actin (red) and cortactin (purple). Histograms represent the mean normalized to control ± SD of (**D**) invadopodia by cell (*n* = 66–88) and (**E**) area of degradation (*n* = 76–94) from 3 independent experiments. (**F**) Confluent cell layers of A375 melanoma cells transiently transfected were wounded, and cells were allowed to migrate for 12 h. (**G**) Histogram represents the mean speed migration normalized to control ± SD from 3 independent experiments; ***, *p* < 0.001; unpaired *t*-test compared to control condition.

The C-terminal FAT domain of FAK interacts with the LD2–LD4 motif of paxillin to recruit FAK to FA [27,28,29]. Therefore, we hypothesized that a peptide containing the LD2–LD4 motif would compete with paxillin for binding to the FAT domain of FAK, and thus potentially inhibit FAK targeting to FAs. To test our hypothesis, we expressed LD2–LD4 GFP peptides in A375 melanoma cells. Immunofluorescence experiments showed that these peptides were present in the cytoplasm and enriched at FAs, where they colocalized with paxillin. These peptides trigger a displacement of the P-Y397 FAK signal from FAs to the cytoplasm, as shown by the 43% decrease in FA/cytoplasm P-Y397-FAK signal ratio (Figure 8A,B). On the other hand, Western blot analysis revealed that LD2–LD4 overexpression did not alter either FAK expression or FAK activity at Tyr397 (Figure S4). Altogether, these results show that inhibiting FAK–paxillin interaction delocalizes FAK from FA while preserving at least part of its activity in the cytoplasm.

On the other hand, we observed by immunocytochemistry that melanoma cells expressing LD2–LD4–GFP displayed a similar number of invadopodia as normal cells (Figure 8C,D). However, using in situ zymography on cells expressing the peptides, a clear reduction in the ECM degrading activity of invadopodia was shown (Figure 8E and Figure S5). We thus analyzed the effect of these peptides in the wound healing model of migration. In cells expressing LD2–LD4–GFP, the migration speed was reduced by more than 50% compared to control cells (Figure 8F,G), in agreement with other studies showing that FAK localization at FAs is necessary to control FA turn-over and thus cell migration [6,7,17,25]. Taken together, our results show that inhibiting FAK–Paxillin interactions may be an efficient strategy to prevent metastasis dissemination by inhibiting both invadopodia-mediated matrix degradation and FA-mediated cell migration.

## 4. Discussion

FAK is involved in many aspects of cancer development, and studies have revealed a clear link between FAK expression and cancer aggressiveness. Therefore, development of strategies to inhibit FAK is of potential therapeutic interest. Inhibitors of the kinase domain of FAK are effective in animal models and have shown some promising cytostatic effects in clinical trials. However, an alternative way to kinase activity inhibitors would be to inhibit the scaffolding function of FAK by targeting the interaction of FAK with its binding partners. As many FAK tasks coincide with the localization of FAK at FAs, we used site-directed mutagenesis or competitive ligands to disrupt FAK–paxillin interaction. We observed that this strategy blocks FAK–paxillin interaction and inhibits both migration and the proteolytic activity of invadopodia in human melanoma cells.

High expression of FAK has previously been reported in several cancer types, such as carcinomas and sarcomas of the breast, stomach, colon, liver, prostate or urinary bladder [30], and can correlate with poorer outcome [31,32,33]. Here, in agreement with previous studies [13], we report high FAK expression in a panel of human melanoma cell lines as compared to normal human melanocytes. These findings prompted us to evaluate the roles of FAK in melanoma progression. First, we did not find any increase in FAK expression level in metastatic melanoma cells compared to in situ melanoma. Although in line with clinical studies [34], these results question the role of FAK in the metastatic cascade. Cancer dissemination is a complex process that requires coordination between adhesion, migration, and matrix degradation. In this study, we observed invadopodia in in situ and metastatic human melanoma cell lines. However, despite invadopodia formation, we found that in situ melanoma cells were not able to degrade the extracellular matrix in clear opposition to metastatic cell lines. Invadopodia initially form precursors enriched in F-actin and cortactin, which then mature to acquire proteolytic activity [11,35]. We suggest that in situ melanoma cells assemble invadopodia precursors. The invadopodia maturation step then occurs during melanoma progression, in agreement with previous studies describing the involvement of Notch signaling pathways in melanoma progression [36] and invadopodia maturation [37].

We also found that inhibiting FAK activity via either siRNA-mediated FAK depletion or treatment of cells with the FAK kinase inhibitor PF-573228 [14] effectively inhibited melanoma cell migration but increased the proteolytic activity of metastatic melanoma. It is not surprising to note that the inhibition of FAK reduces migratory cell potential, given the abundant literature establishing the role of FAK in the dynamics of FAs [6,7]. On the other hand, although we and others have already reported that FAK depletion increases invadopodia activity, it is the first time that such an effect has been observed using a small molecule inhibitor of FAK activity. These observations raised questions about the use of such ATP competitive inhibitors as anticancer treatments. Indeed, this class of small molecules which includes VS-4718 [38], GSK2256098 [39], PF-562271 [40], and defactinib (VS-6063) [41], which are currently in clinical development and have demonstrated some benefits [42], bind to the kinase pocket of FAK, inhibiting FAK *trans*-autophosphorylation, and thus the level of phosphorylation at FAK-Y397. However, using a mutated form of FAK at Y397, we demonstrated here that it is precisely the lack of phosphorylation at this residue that mediates increased invadopodia activity in metastatic melanoma. This increase is due to the release of Src from FAs, owing to the mutation of its FA binding partner, resulting in enhanced phosphorylation of substrates at invadopodia such as cortactin and Pyk2 [11].

These critical observations highlight several interrogations. Firstly, can an alternative strategy to kinase inhibitors be considered to suppress the pro-migratory functions of FAK? We previously found that FAK expression mutated at its paxillin binding sites in FAK^−/−^ fibroblasts reduces adhesion and migration [17]. Here, we show that this mutant, when expressed in FAK-depleted melanoma cells, decreases their ability to degrade the extracellular matrix. This effect is accompanied by reduced localization of the mutant to FAs and increased P-Y397 FAK level in the cytoplasm. Today, growing evidence provides hope for discovering small drug-like molecules that would target PPII interfaces [43]. For the FAK interactome, the search for PPI inhibitors has been pioneered by the group of William Cance. They discovered several small molecules inhibiting either FAK/p53 [15] or FAK/VEGFR3 [16] interactions that displayed anti-cancer effects in pre-clinical in vitro and in vivo studies [44,45]. Thus, as a proof of concept that targeting FAK–paxillin interactions would be of potential therapeutic interest, we tested the effect of a small peptide containing the LD2 and LD4 domain of paxillin. LD2 and LD4 motifs bind to the hydrophobic patch 1 and hydrophobic patch 2 located at the interface of helix 1–4 and helix 2–3 of the FAT domain of FAK [29]. We found that this peptide successfully reduced FAK activity at FAs while retaining substantial activity elsewhere in cells, as reported by Western blot experiments. We have further shown that this peptide is able to reduce both melanoma migration and degradation of the extracellular matrix, which clearly shows the interest of such a strategy to reduce cell invasion.

How FAK stays active when not located at FAs remains obscure. Recent studies have shown that FAK exists in an autoinhibited conformation in the cytoplasm, and the recruitment at FAs induces the formation of FAK dimmers through FERM–FERM interactions [46]. When FAK dimers bind through a basic patch in the FAK FERM domain to PI(4,5)P2, its autoinhibition is released, thereby triggering autophosphorylation of Y397, which may be facilitated by mechanical forces [47,48]. This sequence of activation is difficult to reconcile with FAK activation outside FAs. However, many reports show FAK remaining active in suspended cells or anchorage-independent cancer cells [49]. Recently, active FAK was found to localize with active integrins on endosomes. The mechanism of FAK activation at these sites requires direct binding of FAK to endosomal components. Importantly, paxillin was absent from endosomes, implying that FAK recruitment and signaling are organized differently on endosomes and FAs [50]. Therefore, inhibiting FAK–paxillin interactions may be without effect on endosomal activation of FAK.

Early studies have also shown, using a dimerization assay, that the autophosphorylation of FAK is independent of cell adhesion and does not involve the formation of stable multimeric complexes [51]. Moreover, FAK dimmers through FERM–FERM interactions are able to form in solution [52], and FAK–FAK interactions may exist in the cytosol, as demonstrated by FCS studies [53]. Other FAK activators include Ezrin, which was shown to induce its activation in suspended kidney-derived epithelial LLC-PK1 cells [54]. Changes in pH which result in H58 deprotonation within the FERM domain leading to FAK conformational changes, also enable FAK Y397 autophosphorylation [55].

Finally, it should be also emphasized that, in melanoma cells, a relationship between melanin pigmentation and metastatic phenotype has been documented. Indeed, melanin granules dramatically modify the elastic properties of pigmented melanoma cells, thereby inhibiting their transmigration abilities [56]. Moreover, the change in elastic properties observed in soft substrates correlates with a decrease in P-FAK levels and the suppression of melanogenesis-related genes [57]. In addition, depending on the biophysical context, melanogenesis has been shown to alter both tumor growth and tumor progression [58,59]. Thus, melanin pigmentation is possibly an important factor that should be taken into account when using anti-FAK therapies, because it may regulate the level of active FAK in melanoma cells. Clearly, further studies will be needed to identify the FAK activation mechanism in and outside FAs in these cells.

## 5. Conclusions

In this study, we clearly showed that altering FAK–paxillin interactions with a competitive peptide inhibits cell migration and matrix degradation, contrary to classical inhibitors of the FAK kinase domain. Our current findings demonstrate that FAK represents an attractive therapeutic target in invadopodia-associated cancer progression, and supports further consideration of PPI inhibitors as anti-FAK therapies for clinical development [60].

## Data Availability

Data is contained within the article or Supplementary Materials.

## Supplementary Materials

The following are available online at https://www.mdpi.com/article/10.3390/cancers13081871/s1, Figure S1: MT1-MMP expression is not altered in melanoma cells, Figure S2: FAK expression is increased in metastatic melanoma, Figure S3: FAK inhibition in invasive melanoma cells, Figure S4: LD2-LD4 did not alter FAK expression or phosphorylation level, Figure S5: LD2-LD4 reduced gelatin degradation in WM983B melanoma cells.
