# Supplementary material for: Inhibiting FAK–Paxillin Interaction Reduces Migration and Invadopodia-Mediated Matrix Degradation in Metastatic Melanoma Cells
Source: Cancers (Basel). 2021 Apr 14;13(8):1871. doi: 10.3390/cancers13081871 (PMC8070677; doi:10.3390/cancers13081871)
Supplement: Supplementary file 1 [file cancers-13-01871-s001.pdf]

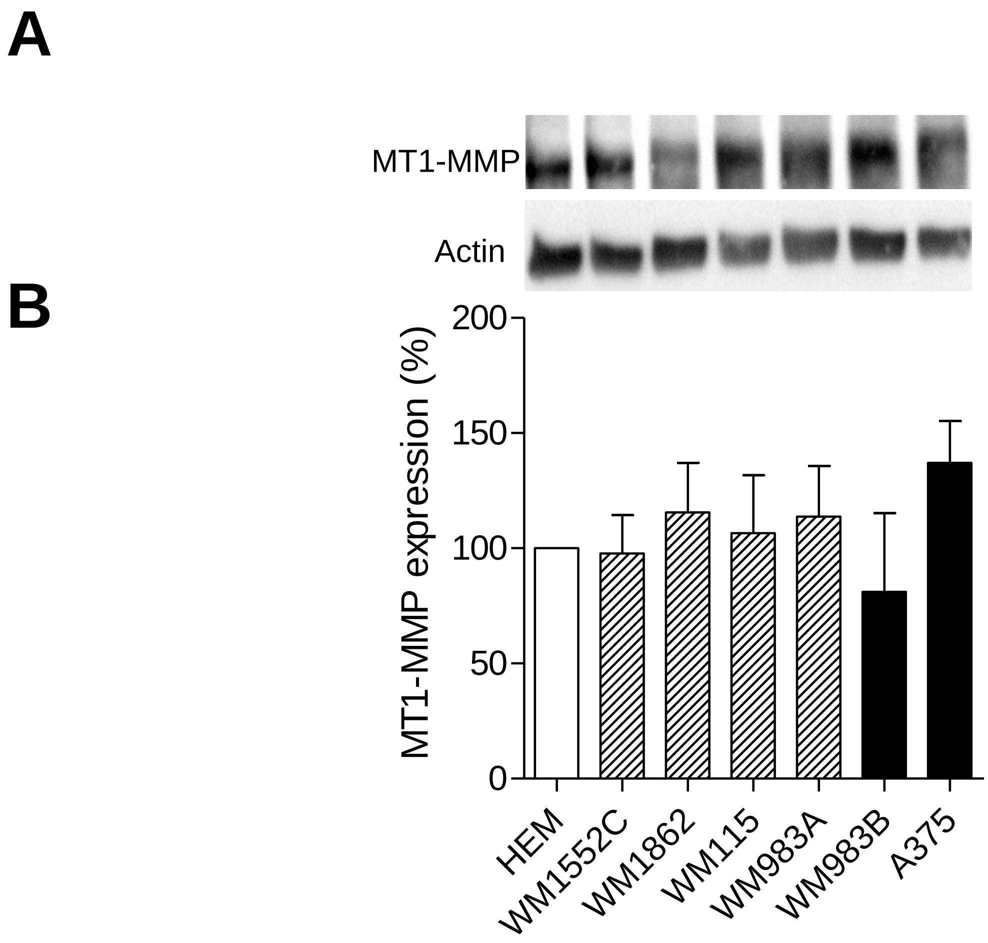


**Supplemental Figure 1: MT1-MMP expression is not altered in melanoma cells (A)** Representative Western blot showing melanoma cells blotted for MT1-MMP and actin. **(B)** Quantification MT1-MMP expression from 3 independent experiments

# Supplemental Figure 1

A

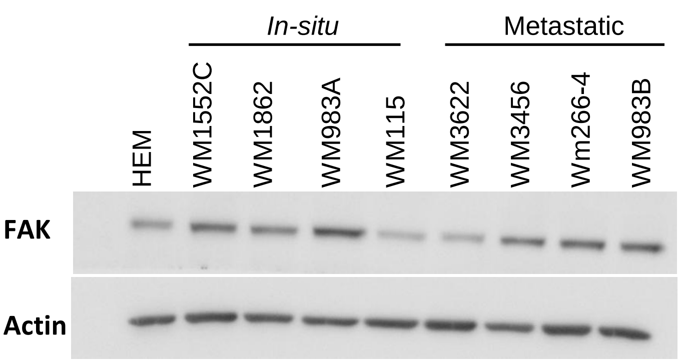


B

| Cell line | FAK Total | Actin | FAK / actin | FAK expression % |
|---|---|---|---|---|
| HEM | 1 843 939 | 3 452 161 | 0,53 | 100,00 |
| WM 1552C | 3 108 530 | 4 472 133 | 0,70 | 130,13 |
| WM 1862 | 2 471 666 | 3 536 463 | 0,70 | 130,85 |
| WM 983A | 3 866 495 | 3 598 082 | 1,07 | 201,18 |
| WM 115 | 1 218 881 | 3 981 287 | 0,31 | 57,32 |
| WM 3622 | 1 190 103 | 4 555 099 | 0,26 | 48,91 |
| WM 3456 | 2 268 356 | 3 182 018 | 0,71 | 133,46 |
| WM 266-4 | 2 788 878 | 4 535 030 | 0,61 | 115,13 |
| WM 983B | 2 779 170 | 4 457 503 | 0,62 | 116,73 |

C

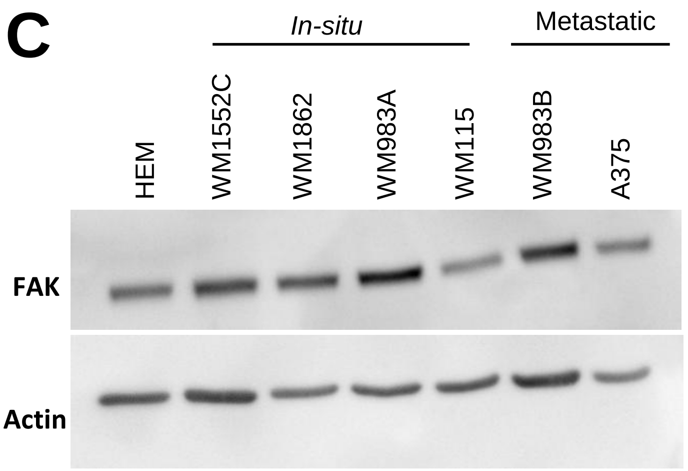


D

| Cell line | FAK Total | Actin | FAK /Actin | FAK expression % |
|---|---|---|---|---|
| HEM | 1 337 049 | 1 595 230 | 0.83 | 100,00 |
| WM 1552C | 1 999 453 | 1 854 770 | 1,07 | 128,62 |
| WM 1862 | 1 629 791 | 1 119 695 | 1,45 | 173,66 |
| WM 983A | 2 276 668 | 1 337 813 | 1,70 | 203,04 |
| WM 115 | 913 669 | 1 315 108 | 0,69 | 82,89 |
| WM 983B | 1 923 129 | 1 836 710 | 1,04 | 124,92 |
| A375 | 954 650 | 869 118 | 1,09 | 131,05 |

E

| | A | B | C | D | E | F | G | H | I | J |
|---|---|---|---|---|---|---|---|---|---|---|
| | HEM | WM1552C | WM1862 | WM983A | WM3622 | WM3456 | WM266-4 | WM115 | WM983B | A375 |
| | Y | Y | Y | Y | Y | Y | Y | Y | Y | Y |
| 1 | 100.00 | 235.96 | 171.40 | 328.06 | 72.12 | 307.64 | 464.21 | 101.21 | 156.50 | |
| 2 | 100.00 | 194.14 | 158.52 | 241.05 | 39.79 | 197.30 | 174.62 | 224.67 | 131.75 | |
| 3 | 100.00 | | 118.05 | | | | 128.83 | 180.74 | 210.40 | |
| 4 | 100.00 | 153.17 | 163.07 | 199.31 | 57.13 | 187.24 | 208.89 | 84.04 | 116.73 | 172.18 |
| 5 | 100.00 | 130.13 | 130.85 | 201.18 | 48.95 | 133.97 | 114.49 | 57.32 | | 125.72 |
| 6 | 100.00 | 128.62 | 173.66 | 203.04 | | | | 82.89 | 124.92 | 131.05 |

F

| | Col. stats | A | B | C | D | E | F | G | H | I | J |
|---|---|---|---|---|---|---|---|---|---|---|---|
| | | HEM | WM1552C | WM1862 | WM983A | WM3622 | WM3456 | WM266-4 | WM115 | WM983B | A375 |
| | | Y | Y | Y | Y | Y | Y | Y | Y | Y | Y |
| 1 | Number of values | 6 | 5 | 6 | 5 | 4 | 4 | 5 | 6 | 5 | 3 |
| 2 | | | | | | | | | | | |
| 3 | Minimum | 100.0 | 128.6 | 118.1 | 199.3 | 39.79 | 134.0 | 114.5 | 57.32 | 116.7 | 125.7 |
| 4 | Maximum | 100.0 | 236.0 | 173.7 | 328.1 | 72.12 | 307.6 | 464.2 | 224.7 | 210.4 | 172.2 |
| 5 | | | | | | | | | | | |
| 6 | Mean | 100.0 | 168.4 | 152.6 | 234.5 | 54.50 | 206.5 | 218.2 | 121.8 | 148.1 | 143.0 |
| 7 | Std. Deviation | 0.0 | 46.11 | 22.84 | 55.08 | 13.72 | 72.91 | 142.5 | 65.69 | 37.88 | 25.43 |
| 8 | Std. Error | 0.0 | 20.62 | 9.325 | 24.63 | 6.859 | 36.45 | 63.74 | 26.82 | 16.94 | 14.68 |

# Supplemental Figure 2

**Supplemental Figure 2: FAK expression is increased in metastatic melanoma.** **(A, C)** Representative western blot of HEM and melanoma cells and their respective quantification **(B, D)**. Cellular extracts were analysed by Western blotting and probed for FAK total and actin. Quantification of FAK expression showing row values **(E)** and statistics **(F)**.

**A**

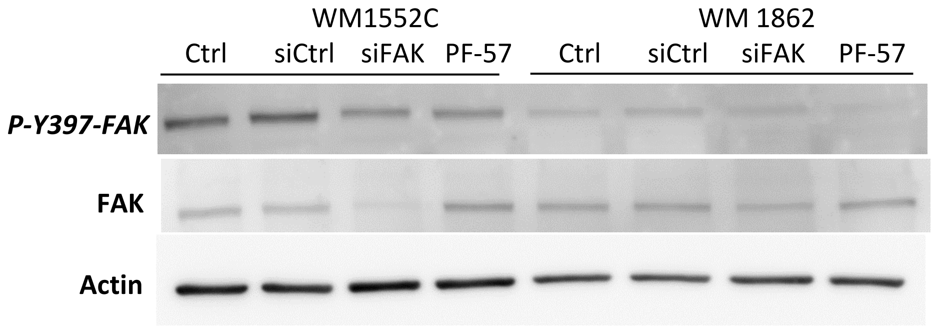


**B**

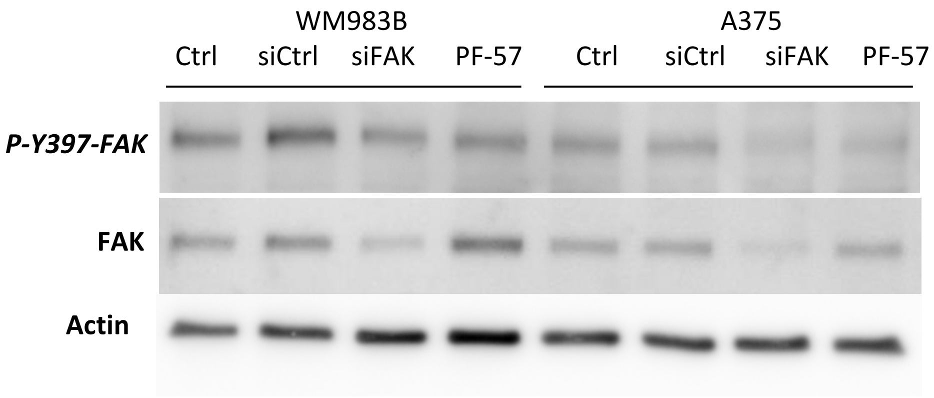


**C**

| Grouped | Ctrl | | | | | | | siCtrl | | | | | | | siFAK | | | | | | | PF-57 | | | | | | |
|---|---|---|---|---|---|---|---|---|---|---|---|---|---|---|---|---|---|---|---|---|---|---|---|---|---|---|---|---|
| | A:Y1 | A:Y2 | A:Y3 | A:Y4 | A:Y5 | A:Y6 | A:Y7 | B:Y1 | B:Y2 | B:Y3 | B:Y4 | B:Y5 | B:Y6 | B:Y7 | C:Y1 | C:Y2 | C:Y3 | C:Y4 | C:Y5 | C:Y6 | C:Y7 | D:Y1 | D:Y2 | D:Y3 | D:Y4 | D:Y5 | D:Y6 | D:Y7 |
| WM1552C | 100.00 | 100.00 | 100.00 | 100.00 | 100.00 | | | | 104.86 | 113.93 | 92.87 | | | | | 33.59 | 46.77 | 21.58 | | | | | 155.43 | 131.00 | 142.61 | | | |
| WM1862 | 100.00 | 100.00 | 100.00 | 100.00 | 100.00 | | | | 103.17 | 88.90 | 112.62 | | | | | 50.63 | 48.60 | 44.24 | | | | | 100.67 | 121.39 | 114.67 | | | |
| WM983B | 100.00 | 100.00 | 100.00 | 100.00 | 100.00 | | | | 99.37 | 81.66 | 83.46 | 98.84 | | | 39.81 | 33.20 | 49.97 | 29.41 | 30.80 | | | 108.57 | | 68.38 | 105.65 | 116.52 | 106.60 | |
| A375 | 100.00 | 100.00 | 100.00 | 100.00 | 100.00 | | | 104.76 | 109.03 | 115.79 | 95.58 | 61.92 | | | 34.26 | 37.54 | 69.18 | 36.64 | 44.24 | | | 106.78 | 100.60 | 127.21 | 82.50 | 99.28 | 93.66 | 112.15 |

**D**

| Row stats | | A | | | B | | | C | | | D | | |
|---|---|---|---|---|---|---|---|---|---|---|---|---|---|
| | | Ctrl | | | siCtrl | | | siFAK | | | PF-57 | | |
| | | Mean | SD | N | Mean | SD | N | Mean | SD | N | Mean | SD | N |
| 1 | WM1552C | 100.000 | 0.000 | 5 | 103.887 | 10.564 | 3 | 33.980 | 12.600 | 3 | 143.013 | 12.220 | 3 |
| 2 | WM1862 | 100.000 | 0.000 | 5 | 101.563 | 11.941 | 3 | 47.823 | 3.265 | 3 | 112.243 | 10.571 | 3 |
| 3 | WM983B | 100.000 | 0.000 | 5 | 90.833 | 9.583 | 4 | 36.638 | 8.455 | 5 | 101.144 | 18.809 | 5 |
| 4 | A375 | 100.000 | 0.000 | 5 | 97.416 | 21.153 | 5 | 44.372 | 14.354 | 5 | 103.169 | 14.207 | 7 |

**E**

| Grouped | Ctrl | | | | | PF-57 | | | | |
|---|---|---|---|---|---|---|---|---|---|---|
| | A:Y1 | A:Y2 | A:Y3 | A:Y4 | A:Y5 | B:Y1 | B:Y2 | B:Y3 | B:Y4 | B:Y5 |
| WM1552C | 100.00 | 100.00 | 100.00 | 100.00 | 100.00 | 31.03 | 27.22 | 35.22 | 33.56 | 45.41 |
| WM1862 | 100.00 | 100.00 | 100.00 | 100.00 | 100.00 | 12.59 | 13.21 | 21.60 | 41.96 | 32.21 |
| WM983B | 100.00 | 100.00 | 100.00 | 100.00 | 100.00 | 46.14 | 56.29 | 55.17 | 46.02 | 54.02 |
| A375 | 100.00 | 100.00 | 100.00 | 100.00 | 100.00 | 26.84 | 32.81 | 25.58 | 29.32 | 58.74 |

**F**

| Row stats | | A | | | B | | |
|---|---|---|---|---|---|---|---|
| | | Ctrl | | | PF-57 | | |
| | | Mean | SD | N | Mean | SD | N |
| 1 | WM1552C | 100.000 | 0.000 | 5 | 34.488 | 6.809 | 5 |
| 2 | WM1862 | 100.000 | 0.000 | 5 | 24.314 | 12.667 | 5 |
| 3 | WM983B | 100.000 | 0.000 | 5 | 51.528 | 5.038 | 5 |
| 4 | A375 | 100.000 | 0.000 | 5 | 34.658 | 13.742 | 5 |

# Supplemental Figure 3

**Supplemental Figure 3: FAK inhibition in invasive melanoma cells**. (**A, B)** Melanoma cells were transfected for 48h with control siRNA or FAK siRNA or treated by PF-573228. Cellular extracts were analysed by Western blotting. Quantification of FAK expression showing row values **(C)** and Mean ± SD **(D)**. Quantification of FAK phosphorylation level showing row values **(E)** and Mean ± SD **(F)**.

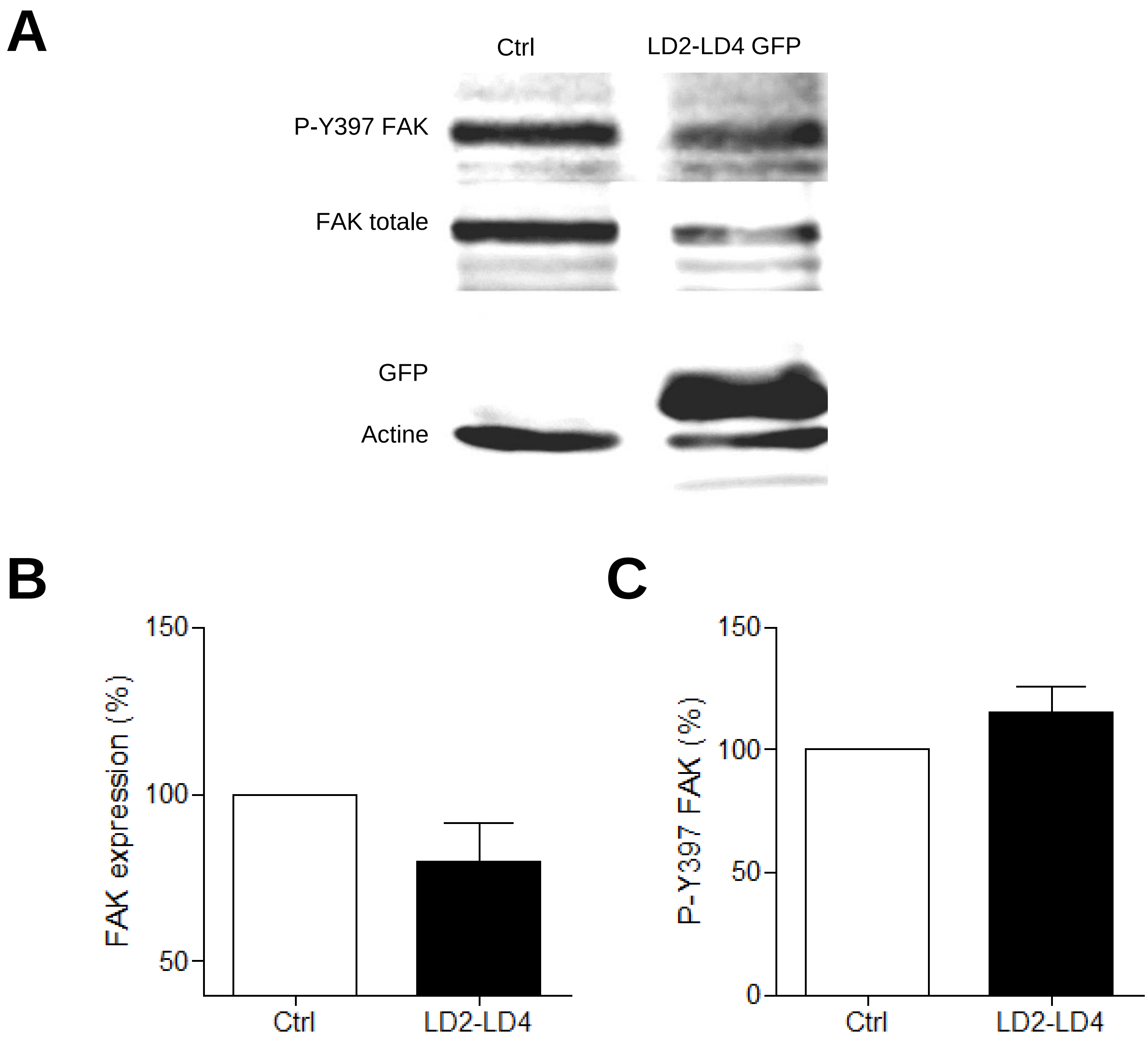


**Supplemental Figure 4: LD2-LD4 did not alter FAK expression or phosphorylation level .** (**A)** Representative western blot showing A375 melanoma cells transfected with LD2-LD4-GFP and blotted for P-Y397 FAK, FAK total, eGFP and actin. **(B)** Quantification of FAK expression and (C) FAK phosphorylation after LD2-LD4-GFP transfection. Histograms represent the Means ± SD from 3 independent experiments

# Supplemental Figure 4

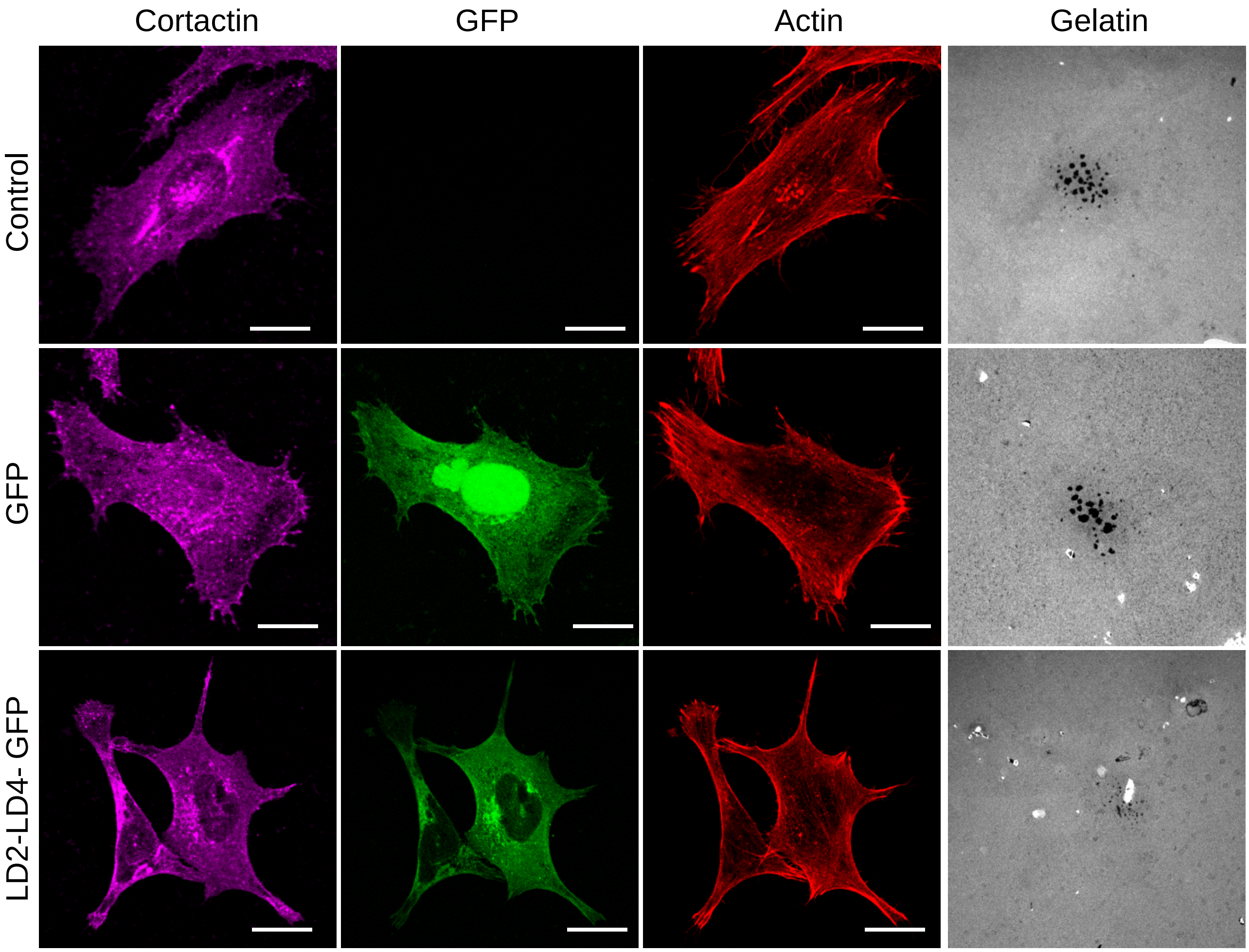


**Supplemental Figure 5: LD2-LD4 reduced gelatin degradation in WM983B melanoma cells.** Representative WM983B melanoma cells transiently transfected with GFP (middle panel) or LD2-LD4 tagged with GFP (lower panel) were plated on Cy3-Gelatin (Gray), fixed, and labelled for actin (Red) and cortactin (Purple).

# Supplemental Figure 5
